# Mapping Global Research Trends on Autism Spectrum Disorder: A Bibliometric Analysis of Pharmacology and Pharmacy Studies

**DOI:** 10.3390/ph19010102

**Published:** 2026-01-07

**Authors:** Gianfranco Sabadini, Angelina Palacios-Muñoz, Isaac E. García, Javier Romero-Parra, Daniel Moraga, Mauricio Soto, Alejandro Vega-Muñoz, Nicolás Contreras-Barraza, Guido Salazar-Sepúlveda, Jaime Mella, Marco Mellado

**Affiliations:** 1 Instituto de Química, Facultad de Ciencias, Universidad de Valparaíso, Av. Gran Bretaña 1111, Valparaíso 2360102, Chile; gianfranco.sabadini@postgrado.uv.cl; 2 Centro de Investigación en Ciencias Odontológicas y Médicas, Facultad de Odontología, Universidad de Valparaíso, Valparaíso 2360004, Chile; angelina.palacios@uv.cl (A.P.-M.); isaac.garcia@uv.cl (I.E.G.); 3 Instituto Milenio Centro Interdisciplinario de Investigación en Neurociencia de Valparaíso, Universidad de Valparaíso, Valparaíso 2381850, Chile; 4 Organic Chemistry and Physical Chemistry Department, Faculty of Chemical and Pharmaceutical Sciences, Universidad de Chile, Olivos 1007, Santiago 7820436, Chile; javier.romero@ciq.uchile.cl; 5 Laboratorio de Fisiología, Departamento de Ciencias Biomédicas, Facultad de Medicina, Universidad de Tarapacá, Arica 1000000, Chile; dmoraga@academicos.uta.cl; 6 Departamento de Química, Universidad Técnica Federico Santa María, Av. España 1680, Valparaíso 2340000, Chile; 7 Laboratorio de Bienestar y Comportamiento Organizacional, Universidad Central de Chile, Santiago 8330507, Chile; alejandro.vega@ucentral.cl; 8 Facultad de Ciencias Empresariales, Universidad Arturo Prat, Santiago 8340232, Chile; 9 Facultad de Ciencias Económicas y Administrativas, Pontificia Universidad Católica de Valparaíso, Valparaíso 2340025, Chile; nicolas.contreras@pucv.cl; 10 Facultad de Ingeniería, Universidad Católica de la Santísima Concepción, Concepción 4090541, Chile; gsalazar@ucsc.cl; 11 Facultad de Ingeniería y Negocios, Universidad de Las Américas, Concepción 4090940, Chile; 12 Centro de Investigación, Desarrollo e Innovación de Productos Bioactivos (CInBIO), Universidad de Valparaíso, Valparaíso 2360102, Chile; 13 Dirección de Investigación, Universidad Bernardo O’Higgins, Santiago 8370993, Chile; 14 Centro de Investigación en Ingeniería de Materiales, Universidad Central de Chile, Santiago 8330507, Chile

**Keywords:** autism spectrum disorder, bibliometric analysis, neurodevelopmental disorders, oxidative stress, synaptic plasticity, translational research, global scientific trends, knowledge mapping

## Abstract

**Background**: Autism spectrum disorder (ASD) represents a major challenge in neurological development research and is receiving increasing attention from the pharmacological and pharmaceutical sciences. Despite this constant growth, there is no document that provides a comprehensive overview integrating publication trends, key contributors, and thematic developments, allowing efforts to be focused on specific areas. **Objective**: To conduct a comprehensive bibliometric analysis of pharmacological research related to ASD published between 2001 and 2025. **Methods**: The database obtained contains 1170 articles indexed in the Web of Science (WoS) database in the JCR Pharmacy and Pharmacology category. Bibliometric indicators such as publication growth, h-index, authorship, institutional and national productivity, and keyword co-occurrence were analyzed using VOSviewer and the laws of Price, Bradford, Zipf, and Lotka. **Results**: A total of 1170 documents were analyzed, showing an exponential increase in pharmacological research related to ASD over the last two decades. The United States, China, and Italy emerged as the most productive countries, while King Saud University, Harvard Medical School, and The Ohio State University were among the leading institutions. The most frequently cited keywords, such as “autism spectrum disorder,” “valproic acid,” “oxidative stress,” and “flavonoids,” revealed a translational approach linking neurobiological mechanisms, redox imbalance, and therapeutic interventions. Contemporary research emphasizes immuno–synaptic interactions, microbiota, and biomarker-guided approaches. **Conclusions**: This study highlights the global expansion and diversification of pharmacological research in ASD. The results underscore a shift toward integrated biological frameworks and precision-oriented strategies, reinforcing the need for interdisciplinary collaboration to advance translational outcomes in ASD therapy.

## 1. Introduction

Autism Spectrum Disorder (ASD) is a neurodevelopmental disorder characterized by persistent difficulties in social communication and social interaction, accompanied by restrictive and repetitive patterns of behavior, interests, or activities [1,2]. Its overall prevalence is close to 1% of the child population [3]. Importantly, ASD is a lifelong condition that accompanies the individual throughout their entire lifespan, even though it is most frequently studied and diagnosed during childhood and adolescence [4]. Recognizing this lifelong trajectory is essential for developing therapeutic, educational, and social strategies that provide sustained support across all stages of life.

The clinical diagnosis, based on criteria defined in the Diagnostic and Statistical Manual of Mental Disorders, Fifth Edition (DSM-5) and the International Classification of Diseases, Eleventh Revision (ICD-11), is also supported by complementary psychometric, neurobiological, and genetic tools. ASD presents considerable etiological and phenotypic heterogeneity, involving genetic, epigenetic, immunological, neurochemical, and environmental alterations [5,6,7,8,9].

Currently, there is no cure for ASD, nor are there any approved pharmacological treatments for its core symptoms. However, early interventions, intensive behavioral therapies, individualized educational support, and the use of medications for comorbidities (such as anxiety, irritability, or ADHD) can significantly improve quality of life [10]. Clinical research is advancing in the evaluation of agents such as oxytocin, propranolol, vasopressin, and bumetanide, with encouraging preliminary results [2].

Despite these advances, several relevant gaps persist: (i) most existing studies analyze the ASD ecosystem as a whole, with less attention to specific disciplinary subfields; (ii) there is a scarcity of analyses focused on the “Pharmacology & Pharmacy” category of Web of Science (WoS), where research on pharmacotherapy, targets, and preclinical–clinical translation is concentrated; (iii) networks and “hotspots” are often described without cohesively integrating classical bibliometric laws (Lotka–Bradford–Price) with modern science maps; and (iv) performance metrics are seldom triangulated with keyword cartographies that allow inference of translational axes between preclinical models, neurobiology, and clinical management [11,12,13,14,15,16].

In this context, the present study conducts a comprehensive bibliometric analysis of pharmacological research on ASD within the “Pharmacology & Pharmacy” category of the Web of Science. Using a structured corpus of 1170 articles, classical bibliometric laws (Lotka, Bradford, and Price) are integrated with science-mapping tools (VOSviewer 1.6.20) to characterize dynamic publication, influential actors, thematic evolution, and translational research fronts. This approach provides a quantitative and reproducible framework to contextualize current pharmacological efforts in ASD and to identify emerging directions relevant to therapeutic development and research prioritization.

## 2. Results and Discussions

Using the Web of Science Core Collection as the data source, a structured bibliometric search was conducted employing the topic query TS = (Autism NEAR/0 Spectrum NEAR/0 Disorders), which retrieved records containing the exact phrase “Autism Spectrum Disorders” in the title, abstract, or keywords. This initial search yielded 63,866 documents. When restricting the results to research articles only, the dataset was reduced to 46,570 records. Subsequently, applying the Journal Citation Reports (JCR) category filter “Pharmacology & Pharmacy” narrowed the corpus to 1170 documents addressing pharmaceutical and pharmacological aspects of ASD. The earliest record in this subset dates back to 2001, while continuous (gap-free) publication begins in 2003, resulting in a total of 1116 documents within the analyzed period.

### 2.1. Trends and Growth in Publications Around the Autism Spectrum Disorder

**Figure 1:**
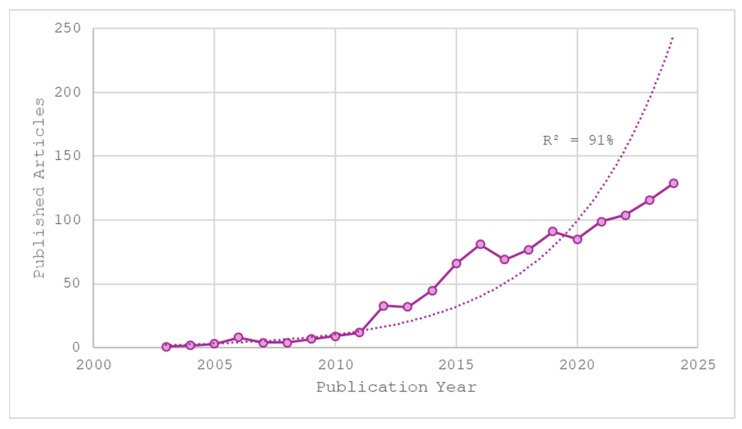
Temporal distribution of publications per year from 2001 to 2024. Solid Line: data on the number of items found in the database. Dashed line: fitting data to an exponential growth curve.

The first records of research on autism from the perspective of pharmacology and pharmacy worldwide date back to 2001, with a pilot study titled “Effect of fluoxetine on regional cerebral metabolism in autistic spectrum disorders” [17]. Since 2003, there has been a sustained trend in publications on this topic, which consolidated in exponential growth until 2024, with a correlation adjustment of 91%, as shown in Figure 1. This behavior reflects the formation of a critical mass of scientific evidence surrounding ASD, highlighting the growing relevance of pharmacology and pharmacy in addressing this condition.

Figure 1 shows that between 2010 and 2020, there was a significant increase in the number of studies, a phenomenon that may be associated with the diagnostic redefinitions introduced by the DSM-5 [18,19], prioritizing autism as a public health issue [20] and the incorporation of new pharmacological approaches [21,22]. In contrast, between 2021 and 2024, there is evidence of a slowdown in scientific production on ASD, which could reflect the disruptive effects of the COVID-19 pandemic on clinical research and the limited results of certain drug trials in addressing core symptoms [23], as well as a redirection of funding towards other therapeutic or technological strategies [24]. However, despite this slowdown, almost half of contemporary studies in this field were concentrated in the period 2020–2024, representing 49.5% of the total and establishing itself as the period of contemporary publications.

### 2.2. Worldwide Collaboration Networks Around the Topic Autism Spectrum Disorder

**Figure 2:**
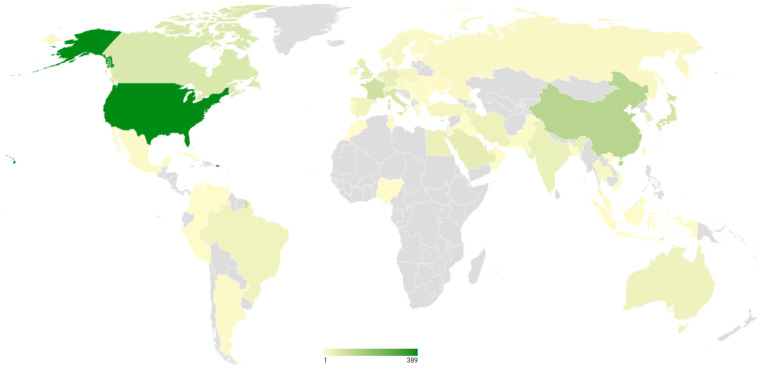
Countries with the greatest contribution to the study of Autism Spectrum Disorder from the perspective of pharmacology and pharmacy.

During the period analyzed (2003–2024), global research on ASD from the perspective of pharmacology and pharmacy shows that the five countries with the highest scientific output in this field are the United States (389 documents), China (130 documents), France (88 documents), Japan (81 documents), and Italy (67 documents), as shown in Figure 2.

In the United States, it is estimated that in the 1970s, the prevalence of ASD was approximately one case per 5000 children, while by 2000, prevalence had risen to one per 500 children [25,26]. In more recent years, the reported incidence has reached levels of one in every 36 children, reflecting a sustained increase in the recognition and diagnosis of this condition [27]. In light of this situation, the National Institutes of Health (NIH) implemented the Autism Centers of Excellence (ACE) program, aimed at promoting research projects focused on deepening understanding of ASD and developing therapeutic interventions [28]. In its most recent renewal, carried out in 2022, the program received USD 100 million in funding to support initiatives until 2026 [29]. In terms of funding entities, the United States Department of Health and Human Services (164 articles), the National Institutes of Health (NIH, 160 articles), the NIH National Institute of Mental Health (NIMH, 62 articles), the NIH Eunice Kennedy Shriver National Institute of Child Health and Human Development (NICHD, 27 articles), and the NIH National Institute of Environmental Health Sciences (NIEHS, 17 articles) together have fully or partially funded 430 scientific publications in the field. In this regard, in 2023, the United States Department of Health and Human Services had a budget of 17.6% of gross domestic product (GDP) for the funding of various agencies such as the NIH, and this budget is expected to increase to 20.3% by 2033 [30], a situation that is consistent with the high number of articles published and funded by the various agencies of the Department of Health and Human Services.

In China, the prevalence of ASD was estimated at 26.5 per 10,000 people in 2016, as reported by Liu Xian [31], rising to 70 per 10,000 inhabitants in 2023 [32]. However, until 2016, there was a marked lack of epidemiological data at the national level, which contributed to both underdiagnosis and undertreatment of this condition [33], although the first institute dedicated to autism in the country, the Beijing Stars and Rain Education Institute for Autism, had already been founded in 1993 [34]. In relation to funded research, it was identified that the National Natural Science Foundation of China (NSFC) fully or partially supported 71 articles during the period analyzed. Other relevant funding agencies were also identified, such as the Beijing Municipal Science and Technology Commission (eight articles), the Natural Science Foundation of Heilongjiang Province (seven articles), the China Postdoctoral Science Foundation (seven articles), the National Basic Research Program of China (five articles), and the National Key Research and Development Program of China (five articles). Together, these agencies provided funding for 108 of the 137 articles identified, reflecting the progressive consolidation of a scientific support ecosystem in the field of ASD in China.

These funding patterns (EE.UU and China) are embedded within national science and health policies that prioritize neurodevelopmental disorders, supported by robust biomedical research infrastructures, long-term grant mechanisms, and dedicated translational centers. Such structural conditions enable continuity across basic, preclinical, and pharmacological research stages, which is reflected in sustained publication output and institutional leadership.

In France, it is estimated that between 1% and 2% of the population is directly affected by ASD, according to the Centre National de la Recherche Scientifique (CNRS) [35]. In this context, the document titled Alternative Report of the French Autism Association to the Committee on the Rights of the Child, prepared by Danièle Langloys, highlights that in February 1989, the first call was made for the “establishment of a movement for the right to educational and non-psychoanalytic care for autistic people,” which culminated in a founding assembly in June of the same year in Lyon. At that meeting, the first “manifesto of parents of autistic children” was drafted [36]. However, it was not until 2005 that the French Federation of Psychiatry (FFP), in collaboration with France’s Haute Autorité de Santé, developed the first Professional Practice Guidelines for the Diagnosis of Autism [37]. About research founded in the field of ASD, the Institut National de la Santé et de la Recherche Médicale (INSERM) was identified as one of the main institutions that supported publications between 2001 and 2024, with a total of 12 articles devoted to studies related to the field of pharmacy and pharmacology. It was followed by the Agence Nationale de la Recherche (ANR) and the Centre National de la Recherche Scientifique (CNRS), each with five articles funded in whole or in part.

In the scientific field, Japan occupies a prominent position worldwide, ranking fourth in the number of publications in Pharmacology and Pharmacy related to ASD. A national study on ASD, conducted between 2009 and 2019, reported that the incidence in Japanese infants exceeds 2.8%, reflecting a sustained increase in diagnosis over the last decade [38]. However, several authors have pointed out that the high prevalence observed could be partially influenced by the diagnostic overlap between ASD and attention deficit hyperactivity disorder (ADHD), especially prior to the publication of the Diagnostic and Statistical Manual of Mental Disorders, Fifth Edition (DSM-5), when clinical criteria tended to favor the diagnosis of ASD over ADHD in comorbid cases [39]. During the period analyzed, 82 scientific articles on Pharmacology and Pharmacy related to ASD were identified with the participation of Japanese institutions. These studies were fully or partially funded by national agencies, notably the Ministry of Education, Culture, Sports, Science and Technology of Japan (MEXT; 48 articles), the Japan Society for the Promotion of Science (JSPS; 47 articles), and Grants-in-Aid for Scientific Research (KAKENHI; 38 articles), reflecting strong government support for research into ASD neuropharmacology in Japan.

In Italy, it is estimated that between 2016 and 2018, the prevalence of ASD reached approximately 13.4 per 1000 children aged 7 to 9, occurring four times more frequently in males than in females [40]. Despite this recent figure, the first scientific article on this topic with authors affiliated with Italian institutions was published in 2009, based on presentations made during the Annual Neurotoxicology Meeting held in Rochester, New York, in October 2008. This work focused on the search for neurotoxicological evidence related to ASD in animal models [41] and was funded by the NIH and the Italian Ministry of Education. In this context, large-scale research consortia have been formed within the European Union to address ASD as a public health issue. Among them is the European Autism Interventions—A Multicenter Study for Developing New Medications (EU-AIMS) project, in which the Università Campus Bio-Medico di Roma is participating, with a contribution from the European Union of EUR 120,682 [42]. Other agencies that have funded research led by Italian teams in the field of pharmacology and pharmacy applied to the treatment of ASD include the Ministry of Education and Research (10 projects), the Italian Ministry of Health (seven projects), and the European Union (five projects).

The predominance of the United States, China, and selected European countries reflects not only sustained public funding but also the alignment of national science policies, dedicated research infrastructures, and long-term translational programs targeting neurodevelopmental disorders. The existence of specialized centers, coordinated funding schemes, and stable institutional networks appears to be a key enabling factor for leadership in pharmacological ASD research, beyond differences in prevalence or population size.

### 2.3. Leading Institutions in the Topic Autism Spectrum Disorder Around the World

During the period analyzed (2001–2024), 1870 institutions worldwide were identified as having participated in at least one scientific article related to ASD in the field of pharmacology and pharmacy. In terms of institutional distribution, there is no significant concentration of scientific production, which shows a relatively dispersed participation among the different academic and research entities. King Saud University stands out as the institution with the highest percentage of publications (1.6%), while the 50 most productive institutions account for approximately one-third of the total contributions (Table S1). Among them, those that have participated in the production of more than 1.0% of the total publications in the area of ASD stand out, which are presented in Table 1.

When comparing the most prolific institutions in the study of ASD from the perspective of pharmaceutical and pharmacological sciences with the countries that concentrate the most scientific production in this area, only institutions in the United States remain within the most productive group. Among them are Harvard Medical School and Massachusetts General Hospital, both located in the state of Massachusetts, along with Columbia University in New York State, all located on the east coast of the United States. In turn, in the center of the country, The Ohio State University (Columbus, Ohio) stands out, while on the west coast, the University of California, Davis (Davis, California) does so. Together, these five institutions account for approximately 5.8% of the total articles published during the period analyzed (2001–2024). On the other hand, there is no direct correspondence between King Saud University (Riyadh, Saudi Arabia), Radboud University Nijmegen (Nijmegen, Netherlands), King’s College London (London, United Kingdom), and the University of Toronto (Toronto, Canada) and the most prolific countries, although together they contribute 4.9% of the total scientific output in the field analyzed.

Analysis of records associated with King Saud University reveals that the most cited article corresponds to a study that evaluated the profile of proinflammatory and anti-inflammatory cytokines and their relationship to the development of ASD. This research demonstrated that cytokines linked to the JAK-STAT signaling pathway play a key role in the immune dysfunction observed in ASD [43]. On the other hand, the most recent publication, corresponding to the year 2024, examined the impact of semaglutide, a glucagon-like peptide-1 (GLP-1) analogue used as an antidiabetic drug, on the behavioral phenotypes of the BTBR autistic mouse model. The results showed that this compound has remarkable therapeutic potential for the treatment of behavioral traits associated with ASD [44]. In both cases, the research reported internal funding from King Saud University. In terms of thematic continuity, the scientific output of this institution shows a transition from the study of the immunological mechanisms involved in ASD to the exploration of innovative pharmacological strategies with therapeutic potential.

In the case of the second most prolific institution, Harvard Medical School, the most cited article corresponds to a 2006 publication, in which a study was conducted on early intervention in ASD, evaluating the efficacy and tolerability of risperidone as a therapeutic alternative. The results indicated that the drug was well tolerated in preschool children over a six-month period; however, only minimal improvement in behavioral symptoms was observed [45]. On the other hand, the most recent article linked to this institution examined the use of psychotropic drugs in children with Down syndrome (DS), based on clinical evidence previously obtained in populations with ASD. The study revealed that almost all patients with DS who were prescribed psychotropic drugs had a concomitant neurodevelopmental (ND) or mental health (MH) condition; however, prescription rates were lower than those observed in children with intellectual disability (ID), ASD, or attention deficit hyperactivity disorder (ADHD). It should be noted that this research did not declare any sources of funding [46]. Considering both studies, Harvard Medical School’s line of research is projected toward the analysis of pharmacotherapy in neurodevelopmental disorders, consolidating a clinical approach focused on evaluating the efficacy and safety of psychotropic treatments in pediatric populations with neuropsychiatric conditions.

The third most prolific institution, The Ohio State University, presents as its most cited article a 2018 publication in which a pilot study was conducted on the administration of probiotics with the aim of improving the quality of life of patients with ASD who presented gastrointestinal symptoms. The results showed a significant improvement in these symptoms, particularly in children with ASD who retained *Lactobacillus* strains. This research was funded by the Health Resources and Services Administration (HRSA) of the U.S. Department of Health and Human Services (HHS), as well as by the Clinical and Translational Science Award granted by the National Center for Translational Sciences [47]. For its part, the most recent article (2020) continues this line of research by evaluating the effectiveness of four doses of psychostimulant medication—through a combination of extended-release and immediate-release methylphenidate—on performance in cognitive tasks, observing a significant improvement in the participants’ performance. This study reported funding from the United States Department of Health and Human Services, through the NIH and the NIMH [48]. Overall, the records from The Ohio State University show a sustained line of research focused on pharmacotherapy for ASD, with an approach aimed at improving cognitive and behavioral functions.

In the case of Massachusetts General Hospital, the fourth most prolific institution in research on ASD in the field of pharmacy and pharmacology, the most cited article corresponds to a 2018 publication, in which a pilot study was conducted on the administration of probiotics in children diagnosed with ASD, carried out in collaboration with The Ohio State University [47]. The most recent article, published in 2024, aimed to establish the relationship between translocator protein (TSPO) and the incidence ratio of ASD by sex (approximately 4:1 in favor of males). The results provided preliminary evidence of increased expression of the mitochondrial protein TSPO in women with ASD, suggesting the presence of female-specific neuroimmunometabolic alterations. This research was funded by the Robert E. and Donna Landreth Fund for the Study of Neuroimmune Interactions in Autism and by the MGH Clinical and Translational Research Unit for Medical Research [49]. Taken together, the findings from Massachusetts General Hospital reveal a thematic evolution that moves from the exploration of probiotic-based clinical interventions to the study of the differential neurobiological mechanisms underlying ASD, integrating gender and neuroimmunology perspectives into pharmacological analysis.

Radboud University Nijmegen ranks as the fifth most prolific institution in the field of pharmacology and pharmacy applied to the study of ASD. Its most cited article, published in 2018, explored the relationship between dorsal striatum volume and levels of N-acetylaspartate (NAA) and glutamate, revealing that alterations in these neurochemical molecules modulate striatal volume in individuals with ASD. The results suggested that decreased NAA could reflect a loss of neuronal integrity or metabolic dysfunction at the cellular level. This study was funded by the UK Research & Innovation (UKRI) Medical Research Council (MRC) [50]. On the other hand, the most recent research, published in 2024, examined the missense variant rs547238576 (R150S) of the oxytocin receptor gene (OXTR), previously identified in Japanese individuals with ASD. The results indicated that this variant enhances OXTR receptor function, which could contribute to the excitatory/inhibitory imbalance characteristic of ASD and other neurodevelopmental disorders. This work was funded by the Ministry of Education, Culture, Sports, Science and Technology (MEXT) and the Japan Society for the Promotion of Science (JSPS) [51]. Overall, the studies reflect a thematic continuity aimed at elucidating the neurochemical and genetic mechanisms underlying ASD, consolidating Radboud University Nijmegen as a leader in the integration of molecular and neurobiological approaches in this field.

The University of California, Davis ranks as the sixth most productive institution in the field of pharmaceutical and pharmacological research on autism spectrum disorder (ASD). Its most cited article, published in 2015, focused on the evaluation of the drug baclofen—a GABAB receptor agonist—in mouse models, considering its two optical antipodes. The results showed that the R-baclofen enantiomer exhibits greater potency than S-baclofen in modulating sociability and reducing repetitive or stereotypical behaviors. This study, funded by the UC Davis MIND Institute, highlighted the therapeutic potential of R-baclofen for improving social deficits associated with ASD [52]. In contrast, the most recent article analyzed alterations in cytokine levels in children with ASD who present gastrointestinal symptoms, observing an increase in IL-15 and a decrease in IL-10. These results suggest an imbalance in the immune response of this subgroup of patients, evidenced by variations in plasma concentrations of circulating cytokines. This research did not declare any sources of funding [53]. Taken together, the studies reflect an institutional trajectory focused on understanding the biological and pharmacological mechanisms underlying ASD, with a sustained emphasis on preclinical experimentation and the search for potential therapeutic targets.

King’s College London ranks as the seventh most prolific institution in research into ASD from a pharmaceutical and pharmacological perspective, even though the United Kingdom is not among the five most productive countries worldwide. Its most cited article—which is also its most recent—is a consensus guideline developed by the British Association for Psychopharmacology, based on clinical evidence on the treatment of catatonia. This document offers recommendations tailored to the needs of specific groups, including children and adolescents, older adults, women in the perinatal period, people with ASD, and patients with relevant medical comorbidities. The guide did not declare any sources of funding [54]. Overall, the work carried out by King’s College London reflects leadership geared toward integrating pharmacotherapeutic evidence into the management of complex psychiatric conditions, including neurodevelopmental disorders such as ASD.

The University of Toronto ranks as the eighth most prolific institution in the field of pharmaceutical and pharmacological sciences applied to the study of ASD. The most cited article, published in 2009, evaluated the effect of sodium valproate on irritability and aggression in children and adolescents with ASD. The results indicated that patients who responded to treatment had higher plasma levels of valproate compared to those who did not respond, suggesting the efficacy of divalproate in managing ASD-associated irritability. This study was funded by US agencies under the NIH, including the National Institute of Neurological Disorders and Stroke (NINDS) and the National Center for Research Resources (NCRR) [55]. For its part, the most recent article (2024) analyzed brain microstates in individuals with ASD and their relationship to the core behavioral characteristics of the disorder, highlighting their potential value as a neurophysiological biomarker. This research was financially supported by the Ministry of Health and Long-Term Care, the Ontario Ministry of Research and Innovation, and the Weston Brain Institute [56]. Considering both studies, there is evidence of thematic continuity that integrates pharmacotherapy and neurophysiology as converging axes in the understanding and treatment of ASD.

Finally, Columbia University ranks ninth among the most productive institutions (≥1% of articles) in the pharmaceutical and pharmacological field of ASD. The most cited article, published in 2016, evaluated the use of arbaclofen in individuals aged 5 to 21 with ASD (phase II study), reporting a significant improvement in the Clinical Global Impression of Severity (CGI) and in the socialization domain of the Vineland II Adaptive Behavior Scales in participants treated with the drug. This study was funded by Roche Pharmaceuticals [57]. In contrast, the most recent article (2024) used a metabolomic approach to examine brain metabolic changes induced by exposure to low doses of pyrethroids during development in adult mice. The results indicate that such exposure alters brain metabolism, which could form the basis for the design of preventive strategies against the neurotoxic effects of development. This research was financially supported by the United States Department of Health & Human Services and the NIH [58]. Based on the contrast between the two studies, it can be observed that Columbia University shows a thematic transition from the clinical evaluation of behavior modulators to the exploration of neurotoxic and metabolic mechanisms associated with ASD.

### 2.4. Prolific Authors and Co-Authorship on the Study of Autism Spectrum Disorder

**Figure 3:**
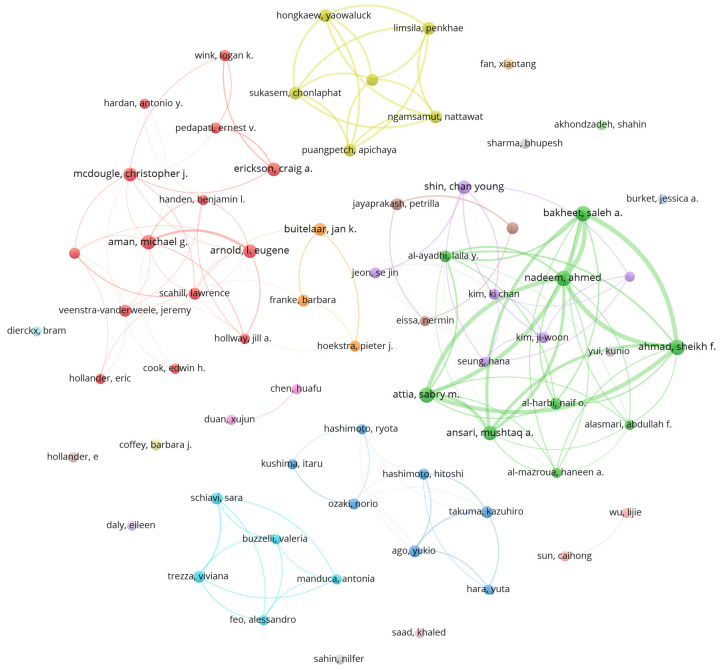
Co-authorship and collaborative networks in the study of autism spectrum disorder in pharmacy and pharmacology field. The colors correspond to the different clusters grouped by the VOSviewer program.

From a total of 1165 articles, the participation of 6492 researchers was identified. Lotka’s law was applied to determine the prolific authors [59,60,61], estimating the square root of 6492 (≈80.57). With this, 68 authors were selected who stand out as the main generators of knowledge in this area. It is important to note that 16 researchers have more than 10 publications related to ASD in the field of pharmaceutical sciences and pharmacology, where 48 have at least four studies and 184 have a minimum of three contributions in this discipline. Among the most active authors, Ahmad, Sheikh F., Bakheet, Saleh A., and Nadeem, Ahmed top the list with 22 papers, followed by Attia, Sabry M. with 20 articles and Aman, Michael G. with 16. Considering the recent consolidation of this research topic, those who have developed more than four related studies are classified as prolific (see Figure 3).

In this study, the h-index was calculated for the analyzed database, which includes articles published between 2001 and 2025, obtaining a value of h = 81 (i.e., 81 articles with at least 81 citations, with a total range of 556 to 81 citations in the Web of Science databases).

**Figure 4:**
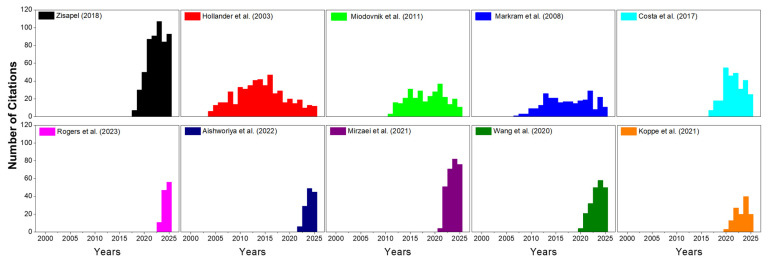
Citation trends for the 10 most cited articles on autism spectrum disorder from 2001 to 2025. The upper panel displays, from left to right, the five most cited authors during 2001–2020, while the lower panel shows those from 2020–2025. The y-axis indicates the number of citations, ranging from 0 to 107—the highest value recorded among the 10 analyzed articles [54,62,63,64,65,66,67,68,69,70].

When comparing this result with the growth trends in publications on ASD described in Section 2.1, most of the articles with the greatest impact belong to the first half of the period analyzed (2001–2020). Among them, the works of Zisapel (2018) stand out [62], followed by Hollander et al. (2003) [63], Miodovnik et al. (2011) [64], Markram et al. (2008) [65], and Costa et al. (2017) [66], presented in descending order according to the number of citations in the ASD area (Figure 4).

These studies address diverse but complementary topics: the first analyzes the relevance of circadian rhythm in neurodevelopmental disorders [62]; the second explores the possible role of oxytocin in the etiology of ASD [63]; the third examines the relationship between exposure to environmental pollutants and the manifestation of this condition [64]; the fourth delves into the neural mechanisms underlying the behaviors characteristic of the autism spectrum [65]; and the fifth links air quality to neurodevelopmental disorders [66].

On the other hand, the most cited articles that make up the h-index within the most recent segment of the analyzed period (2020–2025) show both thematic and methodological diversification (Figure 4). In descending order of citations, the works that stand out are those of Rogers et al. (2023), who developed an evidence-based guide for the management of catatonia associated with ASD [54]; Aishworiya et al. (2022), who prepared a comprehensive update on therapeutic strategies in the treatment of the disorder [68]; Mirzaei et al. (2021) and Wang et al. (2020), who analyzed the role of gut microbiota in nervous system disorders [69,70]; and finally, Koppe et al. (2021), who explored the application of big data tools in psychiatric diagnosis [67].

Taken together, these findings show an evolution in the scientific approach, which has shifted from studies focused on biological and environmental mechanisms (2001–2020) to research aimed at integrating emerging technologies, clinical guidelines, and systemic approaches for understanding and treating ASD in the most recently analyzed period (2020–2025). Combining the research growth and the keywords evolution, early-phase research (2001–2010) was dominated by behavioral pharmacology, environmental exposure models, and monoaminergic hypotheses. In contrast, recent studies (2018–2025) emphasize immuno-synaptic mechanisms, microbiota–gut–brain interactions, and biomarker-guided therapeutic strategies, reflecting a maturation toward integrative and systems-level approaches.

### 2.5. Nucleus of Journals in the Autism Spectrum Disorder Field

The most influential journals in the field of pharmacy and pharmacology research on ASD were identified using Bradford’s law, which states that a small number of journals account for most of the relevant scientific output on a specific topic. In addition, it was found that the rest of the scientific output is dispersed across scientific contributions that are equally significant, although less frequent. The dataset exhibited an h-index of 81, with an exponential annual growth trend particularly pronounced after 2015. Bradford’s law revealed a small core of high-impact journals accounting for a disproportionate share of total citations, followed by two broader peripheral zones.

In this context, research related to ASD (n = 1170 articles) was analyzed according to the scientific journals in which the articles were published (Table 2). This table contains the journals that account for the top third (ordered from highest to lowest contribution) and correspond to the core of research in this field.

Considering that the research topic corresponds to ASD, it can be approached from different fields of knowledge. Table 2 lists the most relevant journals in the field of pharmacology and pharmacy. Among them are the Journal of Child and Adolescent Psychopharmacology, Progress in Neuro-Psychopharmacology & Biological Psychiatry, Neuropsychopharmacology, and Annales Medico-Psychologiques, published by Mary Ann Liebert, Elsevier, Springer Nature, and Masson Éditeur, respectively. It is important to note that this set of journals does not include those associated with publishers that have a high publication rate and short review and production times, such as MDPI (Biomedicines, which contributes 3.86%) and Frontiers (Frontiers in Pharmacology, which contributes 2.15%). It should be noted that these publishers concentrate a significant volume of publications in other areas, such as studies on antioxidants [71]. In this same context, the publisher with the highest proportion of publications is Elsevier, which accounts for almost half of this select group of journals recognized as the most influential in research on ASD in the field of pharmacology and pharmacy.

In terms of the prestige of the journals with the highest number of publications, three of the five are in the Q1 quartile and correspond to internationally renowned publishers such as Elsevier and Springer Nature. Likewise, one journal belongs to the Q2 quartile, published by Mary Ann Liebert, while it is striking that this group also includes a journal classified in the Q4 quartile, published by Masson Éditeur.

The impact factor and quartile assigned to each category appear to be closely related to editorial objectives. Thus, the three Q1 journals focus their editorial line on publishing studies with a marked experimental emphasis on biological and neuroscientific sciences, areas that form the basis for the design of new drugs and therapeutic strategies [72,73,74]. In contrast, the journal ranked in the Q2 quartile, Journal of Child and Adolescent Psychopharmacology (Mary Ann Liebert), favors articles focused on the clinical approach and the implications of public policies for the treatment of psychological disorders [75]. Finally, the Q4 journal Annales Medico-Psychologiques focuses on the diagnosis and treatment of mental illnesses, which explains its more clinical than experimental orientation [76].

When comparing the five most influential journals presented in Table 2, only Neuropsychopharmacology (Springer Nature) is published under the open access model. Unlike subscription journals, open access publications tend to have shorter review times, with a reported average of 3 days for the first editorial decision and 98 days until final publication [72]. In contrast, the Journal of Child and Adolescent Psychopharmacology presents an estimated time of 40 days for the first decision [75], a value similar to that reported by Progress in Neuro-Psychopharmacology & Biological Psychiatry [73].

In terms of costs, the article processing charge (APC) in the journal Neuropsychopharmacology amounts to EUR 4190, which is considerably higher than that charged by publishers such as MDPI and Frontiers: approximately 1.6 times more expensive than Biomedicines (MDPI) and 1.33 times more expensive than Frontiers in Pharmacology [77,78].

Citation analysis indicates that landmark publications addressing oxytocin signaling, valproic acid-based models, immune dysregulation, and oxidative stress have played a pivotal role in shaping the field. Highly cited journals within pharmacology and neuroscience acted as hubs for methodological and conceptual consolidation, driving subsequent thematic diversification.

### 2.6. Analysis of Keyword Clusters

**Figure 5:**
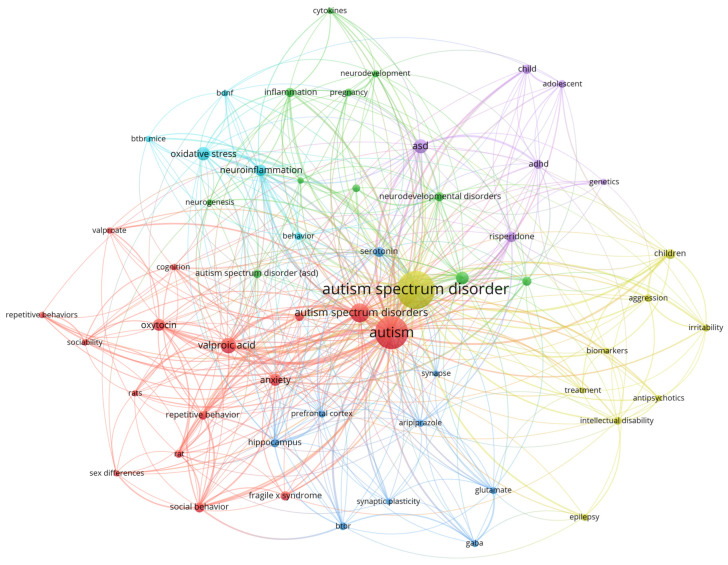
Author keywords and their relations in autism spectrum disorder studies in the pharmacy and pharmacology research field. The colors correspond to the different clusters grouped by the VOSviewer program.

The identification of keywords associated with research on ASD in five-year intervals was carried out by applying Zipf’s law. This principle establishes that the frequency of occurrence of a term is inversely proportional to its position within a list ordered according to its use. In this sense, the analysis allows us to recognize emerging concepts and approaches in pharmacological and pharmaceutical research related to ASD, as presented in Figure 5.

The different clusters of related keywords are presented as different groups in different colors. The first and largest cluster corresponds to the red cluster with 15 relevant terms: among them, “autism/autism spectrum disorders”, “valproic acid/valproate”, “rats/rat”, “repetitive behavior(s)”, “social behavior/interaction”, “sociability”, “oxytocin”, “sex differences”, “cognition”, and “fragile x syndrome”. This cluster captures the experimental tradition in preclinical models focused on ASD core behaviors and social modulators. The valproic acid–rat(s) subgroup articulates a robust axis of environmental modeling of ASD (social and repetitive phenotypes, circuit changes), widely validated in rats and mice, with value for pharmacological screening [79,80,81]. The presence of oxytocin and social terms (sociability, social interaction) reflects a translational interest in improving communication and reciprocity; however, recent randomized controlled trials (RCTs) in pediatric populations have not shown clinically relevant benefits versus placebo, suggesting the need to reorient the hypothesis or better subtype study participants [82,83,84]. The mention of sex differences points to risk/diagnostic biases and the need for sex-stratified designs in models and trials. The co-occurrence of fragile-x syndrome suggests that some mechanisms altering synaptic plasticity (e.g., long-term potentiation, LTP; long-term depression, LTD; local protein synthesis; and excitation/inhibition balance, E/I) are shared by syndromic and non-syndromic ASD.

The second group of terms corresponds to the green cluster that include nine keywords: “autism spectrum disorder”, “cytokines”, “inflammation”, “microglia”, “gut microbiota”, “neurodevelopment/neurogenesis”, “pregnancy”, and “schizophrenia”. This cluster represents an “immune–microbial–neurodevelopmental” axis. Neuropathological and biomarker evidence shows microglial activation and neuroinflammation in the cerebellum and cortex of individuals with ASD [85,86]. In parallel, the pregnancy/maternal immune activation topic links gestational infection/inflammation with neuropsychiatric phenotypes (ASD and schizophrenia), reinforcing the neurodevelopmental character of the cluster [87,88]. The gut microbiota component encapsulates the gut–brain pathway (barrier and microbiota alterations, behavioral rescue in maternal immune activation [MIA] models), opening probiotic and dietary therapeutic avenues that are still incipient [89]. These thematic shifts are consistent with contemporary precision-neuroscience paradigms, which emphasize biologically stratified cohorts, biomarker-informed interventions, and multi-system integration in neurodevelopmental disorders.

The following keyword cluster is the blue colored one that includes nine relevant terms: “GABA”, “glutamate”, “serotonin”, “synapse”, “synaptic plasticity”, “hippocampus”, “prefrontal cortex”, “BTBR”, and “aripiprazole”. The GABA–glutamate–synapse triad represents the E/I hypothesis as a unifying mechanism for several ASD alterations, supported by models and circuit studies [90,91,92]. The BTBR phenotype provides an idiopathic model with social deficits and anatomical–functional changes, useful for exploring plasticity and therapies that correct E/I imbalance [93]. The inclusion of serotonin refers to the hyperserotonemia endophenotype (~25% of cases) and its modulatory relevance for development and social cognition [94].

The yellow cluster shows nine terms: “children”, “biomarkers”, “irritability”, “aggression”, “antipsychotics”, “treatment”, “intellectual disability”, “epilepsy”, and “autism spectrum disorders (ASD)”. This cluster adds the clinical dimension (pediatric population), the search for biomarkers, and therapeutic targets for non-core symptoms. Risperidone and aripiprazole hold FDA approval for irritability associated with autistic disorder (aggression, self-injury, tantrums), serving as a therapeutic bridge to the synaptic and immune clusters [95,96]. The co-occurrence of epilepsy underscores the high prevalence of seizures in ASD (pooled estimate 13%; heterogeneous by age and intellectual disability [ID]) and the need to stratify by epileptogenesis and by the use/response to antiseizure medications (ASMs) or other anticonvulsant therapies [97].

Following is the violet cluster, which displays four relevant terms: “ADHD”, “adolescent/child”, “genetics”, and “risperidone”. This cluster suggests a pediatric psychopharmacological focus with a genetic component and high ASD + ADHD comorbidity. Recent syntheses place ADHD prevalence in ASD in the 30–50% range (depending on age and method), with genetic overlap and shared executive-function profiles [98,99,100]. The presence of risperidone reflects clinical practice in concomitant irritability/aggression and the link to cluster 4 [96].

Finally, the cyan-colored cluster presents as relevant terms five keywords: “BDNF”, “oxidative stress”, “neuroinflammation”, “behavior”, and “BTBR mice”. The oxidative stress–neuroinflammation–BDNF triad suggests routes of synaptic vulnerability and altered plasticity. Blood meta-analyses support oxidative alterations (e.g., reduced GSH and GPx; increased GSSG) and higher peripheral BDNF levels in children with ASD, albeit with between-study and subgroup heterogeneity [101,102]. The link with BTBR mice supports a mechanistic translational interpretation with measures of plasticity and oxidative stress in models.

#### 2.6.1. Key Bridges and Interconnections in the Network

The Figure 5 visualization shows high centrality of “autism spectrum disorder/autism” as a hub anchoring dense connections toward four principal axes. The co-occurrence network of keywords reveals these interconnected thematic clusters, which encapsulate the current research landscape on Autism Spectrum Disorder from pharmacological and neurobiological perspectives. Together, they illustrate the transition from preclinical behavioral models to synaptic, immune, and clinical-therapeutic frameworks, reflecting an increasingly integrated approach to understanding and managing the disorder.

Social–animal model axis (red cluster): valproic acid—rats—repetitive behavior/sociability/oxytocin, with thick edges integrating behavioral phenotypes and pro-social modulators.

Synaptic axis (blue cluster): synapse/synaptic plasticity and hippocampus/prefrontal cortex link to GABA/glutamate and to drugs (Aripiprazole), bridging clinical topics and circuits [92].

Immune axis (green/cyan clusters): cytokines/inflammation/microglia connect with neuroinflammation and oxidative stress (cyan), articulating a cross-cutting biological pathway that also touches pregnancy (gestational risks) and biomarkers [86,103].

Clinical–therapeutic axis (yellow/violet): irritability/aggression/children with antipsychotics (Risperidone, Aripiprazole) and ADHD, evidencing translation from circuits and immunity toward pediatric symptomatic management [100].

#### 2.6.2. Trends and Shifts in Focus

In recent years, research on ASD has shifted from behavioral approaches toward a more integrated understanding of its biological underpinnings. This paradigm shift has promoted the use of both animal and clinical models to explore the neuroimmune, oxidative, and synaptic mechanisms involved in the disorder’s pathogenesis. The following section summarizes three emerging axes that illustrate this conceptual and methodological evolution.

From behavior to integrated biology: the weight of the VPA/BTBR model dyad coexists with a shift toward immune and redox mechanisms (microglia, cytokines, oxidative stress) and circuit-level mechanisms (E/I, GABA, glutamate), consistent with neurodevelopmental hypotheses and E/I instability [86,92].

Translational recalibration: the persistence of oxytocin alongside negative clinical evidence demands subtyping (biomarkers, age, sex) and prioritization of targets that modulate E/I or inflammation, without abandoning pro-social objectives [84].

Complex pediatric clinic: co-occurrence with ADHD and epilepsy suggests differentiated clinical trajectories that require stratified designs (by ID, age, sex, and comorbidities) and endpoints beyond irritability [99].

#### 2.6.3. Forward-Looking Implications for the Research Agenda

Building on the observed thematic structure, several forward-looking implications emerge for shaping the future research agenda on ASD. These recommendations emphasize the need for integrative and biologically informed frameworks that connect neural circuitry, immune pathways, and clinical heterogeneity. The following points outline key directions to enhance translational value, precision, and conceptual coherence across ASD research.

Multi-axis integration (synapse–immunity–microbiota): prioritize studies that concurrently measure E/I (e.g., MRS GABA/glutamate), immune signatures (cytokines, CSF/glial cells), and microbiota features in longitudinal pediatric cohorts; leverage MIA/VPA models for back-translation [88,89].

Biology-guided subtyping and therapeutic response: assess whether redox/inflammatory profiles (GSH, GSSG; IL-6, TNF) and/or BDNF predict response to E/I modulators or anti-inflammatory agents; prior meta-analyses justify biomarker-guided trials [101,102].

Comorbidities as course modulators: incorporate stratification by ADHD and epilepsy in efficacy and safety endpoints (pharmacokinetics, interactions, neurophysiology), given their high prevalence and functional burden [97,100].

Semantic harmonization: reduce noise from synonyms/duplicates (autism spectrum disorder, autism spectrum disorders [ASD]).

Accumulating evidence indicates that immune dysregulation, oxidative stress, and synaptic alterations interact closely in the pathophysiology of ASD. Meta-analyses have consistently reported elevated circulating pro-inflammatory cytokines, including IL-6, TNF-α, and IL-1β, supporting the presence of systemic immune activation in ASD [104,105]. In parallel, disturbances in the glutathione redox system (GSH/GSSG) reflect impaired antioxidant capacity and have been implicated in ASD-related neurobiological vulnerability [106]. Altered peripheral levels of brain-derived neurotrophic factor (BDNF), a key regulator of synaptic maturation and plasticity, have also been reported in children with ASD, underscoring its relevance as a neurodevelopmentally important molecule linking synaptic and immune-related processes [107,108]. Experimental models further support a mechanistic role for BDNF dysregulation in autistic-like phenotypes [109]. Together, these convergent biological pathways highlight candidate mechanisms for disease heterogeneity and provide a framework for future longitudinal studies aimed at evaluating biomarker-informed stratification and treatment response in pediatric ASD.

Long-term pharmacological management of irritability in pediatric ASD commonly involves atypical antipsychotics such as risperidone and aripiprazole [110]. While ASD is characterized by baseline immune dysregulation and oxidative stress, direct longitudinal studies assessing whether chronic antipsychotic exposure modifies neuroimmune or redox biomarkers in children with ASD are still scarce. However, converging experimental and clinical evidence indicates that atypical antipsychotics may influence mitochondrial bioenergetics and metabolic resilience, supporting biological plausibility for interactions with oxidative and immune pathways during critical periods of neurodevelopment [111,112,113,114]. Future longitudinal studies integrating inflammatory cytokines, redox markers, and neurodevelopmental outcomes will be required to disentangle treatment-related effects from disease-intrinsic alterations.

Recent systems-level and multi-omics studies in ASD have begun to provide insight into the convergence of immune, metabolic, microbial, and transcriptomic alterations associated with the disorder. Although most investigations analyze individual or partially integrated omics layers rather than fully unified multi-omics datasets, these approaches consistently highlight dysregulation of pathways related to oxidative metabolism, mitochondrial function, inflammatory signaling, and gut–brain interactions [104,113,115]. Importantly, similar molecular pathways can be experimentally interrogated in preclinical models, supporting the conceptual utility of integrative frameworks for translational research. However, fully integrated, longitudinal multi-omics studies directly linking these molecular signatures to clinical severity or therapeutic response in ASD remain scarce, underscoring a key priority for future translational investigations.

Single biomarkers frequently overlap across neuropsychiatric conditions, limiting their diagnostic utility. Accordingly, increasing attention has been directed toward multimodal biomarker panels integrating inflammatory cytokines (e.g., IL-6, TNF-α), oxidative stress indices (e.g., GSH/GSSG) [104,113], and neurotrophic factors (e.g., BDNF) as tools for biological stratification in ASD rather than categorical diagnosis. Meta-analytic evidence supports altered pro-inflammatory cytokines in ASD [105] and redox imbalance involving glutathione-related pathways, while peripheral BDNF alterations in children with ASD have been summarized in systematic reviews and meta-analyses [101,116]. Notably, inflammatory and oxidative-stress pathways are also implicated in major depressive disorder (MDD), underscoring their transdiagnostic nature and limiting the specificity of single markers [117,118]. Cross-disorder evaluations further indicate that the reproducibility and specificity of inflammatory biomarkers vary across diagnoses, supporting the need for composite, developmentally informed panels and longitudinal validation [119,120]. Although redox and inflammatory pathways are shared across ASD and MDD, ASD-focused research emphasizes neurodevelopmentally relevant redox regulation (e.g., NRF2-linked antioxidant programs), whereas MDD literature highlights oxidative stress signatures that may vary with illness stage and clinical context; direct head-to-head multi-omics comparisons remain scarce [118,121].

Early identification of ASD risk based on biological indicators remains a major challenge. To date, no biomarkers have been clinically validated to reliably predict ASD before symptom onset; however, several biological pathways have emerged as promising targets for longitudinal research. In particular, redox imbalance, including altered glutathione metabolism and increased oxidative derivatives, has been consistently implicated in ASD pathophysiology and may exert effects during early neurodevelopmental windows [113]. In parallel, neurotrophic signaling pathways, such as those involving brain-derived neurotrophic factor (BDNF), play a critical role in synaptic maturation and circuit refinement, and experimental models suggest that their dysregulation may contribute to neurodevelopmental vulnerability [109]. Carefully designed prospective birth and infancy cohorts integrating oxidative, inflammatory, and neurotrophic measures will be essential to determine whether these pathways can ultimately inform early risk stratification and personalized intervention strategies in ASD.

Taken together, these thematic patterns underscore the need for future ASD research to move toward more integrated, biology-driven frameworks.

#### 2.6.4. Emerging Biological Targets and Lead Pharmacological Strategies in ASD

The bibliometric patterns identified in this study reveal a progressive shift from descriptive and behavioral research toward biologically grounded and translationally oriented pharmacological investigations in ASD. Keyword co-occurrence and clustering analyses consistently highlight several convergent biological targets that currently dominate the pharmacology- and pharmacy-focused literature.

One prominent axis corresponds to synaptic and excitation/inhibition (E/I) imbalance, involving dysregulation of glutamatergic and GABAergic neurotransmission [86,92,122]. This framework is strongly represented in preclinical and clinical studies exploring agents such as bumetanide, aripiprazole, and modulators of synaptic plasticity, reflecting efforts to restore circuit-level homeostasis [68]. Closely related, oxytocin and vasopressin signaling pathways emerge as recurrent targets, particularly in studies addressing social cognition and affiliative behaviors, although recent clinical evidence suggests the need for biologically informed subtyping to optimize therapeutic response [63].

A second major translational front is centered on immune and neuroinflammatory mechanisms, including microglial activation, cytokine signaling, and oxidative stress. The strong bibliometric association between valproic acid-based animal models, redox imbalance, and ASD-related phenotypes underscores the relevance of oxidative stress pathways as both mechanistic drivers and potential therapeutic entry points [65,79]. In this context, antioxidant and redox-modulating compounds, as well as agents targeting immune dysregulation, are increasingly explored as adjunct or precision-oriented interventions [123,124].

Finally, contemporary trends point toward system-level approaches, notably the gut–brain axis and microbiota-related interventions [69,70]. The growing visibility of probiotics and microbiome-modulating strategies reflects a broader conceptual transition toward integrated biological models that link metabolic, immune, and neural processes. Together, these findings indicate that current pharmacological research in ASD is converging on a limited but biologically coherent set of targets, providing a rational basis for future drug development, biomarker-guided trials, and translational prioritization.

Future pharmacological ASD research would benefit from integrating artificial intelligence–assisted drug discovery, neuroimmune-targeted interventions, and multi-omics biomarker platforms. Addressing the gap between publication volume and translational impact remains a critical priority.

## 3. Materials and Methods

Based on a dataset extracted from the Core Collection of Web of Science (WoS, including the editions Science Citation Index Expanded (SCI—Expanded), Social Sciences Citation Index (SSCI), Arts & Humanities Citation Index (AHCI), Conference Proceedings Citation Index—Science (CPCI—S), Conference Proceedings Citation Index—Social Science & Humanities (CPCI—SSH), Book Citation Index—Science (BKCI—S), Book Citation Index—Social Science & Humanities (BKCI—SSH), and Emerging Sources Citation Index (ESCI)) on 11 August 2025, with the thematic search vector on Innovative Behavior (TS = (Autism near/0 Spectrum near/0 Disorders)), the thematic search tag TS performed a simultaneous search on the following fields: title, keywords, author, abstract, and Keywords Plus^®^ [125]. In addition, the following inclusion criteria of the sample were included: Document types = Article, Web of Science Categories Pharmacology and Pharmacy. Then, based on the “Guidelines for advancing theory and practice through bibliometric research” [126], both performance analysis and science mapping were performed. For performance analysis, the bibliometric laws of Price [127,128], Lotka [59,61], Bradford [129,130], and Hirsch’s index [131,132] were used, and science mapping focused on co-authorship analysis using VOSviewer software, version 1.6.20, Centre for Science and Technology Studies, Leiden University (details in Table 3).

### 3.1. Bibliometric Laws Used

Price’s Law: Describes the growth of scientific literature, proposing that research output in a field increases exponentially over time until reaching a saturation phase, reflecting the maturation and consolidation of a research domain [127,128].

Lotka’s Law: States that scientific productivity follows an inverse square distribution, where a small number of authors produce most publications, while the majority contribute only one or a few articles within a given field [59,61].

Bradford’s Law: Explains the dispersion of scientific articles across journals, identifying a small core of highly productive journals and successive peripheral zones, with increasing numbers of journals contributing fewer articles [129,130].

Zipf’s Law: Establishes that the frequency of a word is inversely proportional to its rank in a frequency list, enabling the identification of dominant and emerging keywords within a scientific corpus [129].

Hirsch’s index: Measures both productivity and citation impact, defined as the number h of publications that have each received at least h citations, balancing quantity and influence of scientific output [131,132].

### 3.2. Eligibility Criteria

Articles were included if they were (i) indexed in the Web of Science Core Collection, (ii) classified under the Pharmacology & Pharmacy category of the Journal Citation Reports, (iii) published between January 2001 and July 2025, and (iv) explicitly addressed autism spectrum disorder in relation to pharmacological mechanisms, drug targets, experimental models, or therapeutic interventions.

Records were excluded if they were unrelated to ASD, focused exclusively on educational, sociological, or non-pharmacological interventions, or lacked a clear pharmacological or pharmaceutical component. Duplicate records were automatically removed using the WoS export filtering system and manually verified. Borderline articles were retained only when a clear pharmacological dimensions such as drug exposure, molecular targets, pharmacotherapy, or experimental intervention—was identifiable in the title, abstract, or keywords.

This study follows established methodologies for bibliometric research rather than PRISMA guidelines, which are specific to systematic reviews and meta-analyses.

## 4. Conclusions

This bibliometric study provides a comprehensive overview of the evolution of research on autism spectrum disorder (ASD) within the pharmacology and pharmacy domain over the period 2001–2025. The analysis reveals a sustained and exponential growth in scientific output, consistent with the observed exponential increase in publication output (Figure 1), reflecting the growing prominence of pharmacological and pharmaceutical approaches in addressing the biological complexity and therapeutic challenges of ASD.

The results identify a geographically concentrated yet internationally influential research landscape, led primarily by the United States, China, and key European countries. Notably, institutions such as King Saud University, Harvard Medical School, and The Ohio State University emerge as central hubs of knowledge production, indicating that institutional leadership is not strictly dependent on national-level productivity, as supported by institutional output patterns (Table 1, Figure 2) but also reflects sustained strategic investment and consolidated research programs.

Keyword co-occurrence and thematic mapping demonstrate a translational orientation of the field, as evidenced by keyword co-occurrence clusters linking experimental models, oxidative stress, and immune-related mechanisms (Figure 5). Experimental models based on valproic acid, together with recurrent references to oxidative stress and redox imbalance, underscore the prominence of preclinical paradigms linking neurodevelopmental disruption, synaptic plasticity, and molecular pathophysiology. At the same time, the growing visibility of terms related to immunological mechanisms, microbiota, and biomarker-guided strategies indicates a progressive shift toward integrated and systems-level frameworks in ASD pharmacological research.

From a scientific impact perspective, individual productivity analyses highlight the role of stable research groups with long-term thematic continuity, reinforcing the importance of institutional environments that support sustained inquiry and interdisciplinary collaboration. Collectively, these findings suggest a progression of the field from predominantly mechanistic and model-driven studies toward more integrative and translational research agendas (Figure 5).

Despite its contributions, this study has limitations inherent to bibliometric approaches. The reliance on indexed databases may exclude relevant non-indexed or regional publications, and the quantitative nature of the analysis precludes direct assessment of methodological rigor or clinical efficacy. Moreover, bibliometric trends must be interpreted considering the still incomplete understanding of ASD etiology, which arises from complex interactions among genetic, environmental, and epigenetic factors. Recognizing this gap between publication volume and translational impact is essential for guiding future pharmacological research.

Overall, this work offers an evidence-based mapping of research trajectories in ASD pharmacology and pharmacy, providing an evidence-based and structured overview that may support researchers, clinicians, and funding agencies in identifying priority areas and collaboration opportunities.

## Figures and Tables

**Table 1:** Around the world, institutions that contribute most to research related to autism spectrum disorder focused on the pharmacology and pharmacy field.

Entry	Institution	Country	Percentage of Contribution
1	King Saud University	Saudi Arabia	1.6
2	Harvard Medical School	United States of America	1.4
3	The Ohio State University	United States of America	1.2
4	Massachusetts General Hospital	United States of America	1.1
5	Radboud University Nijmegen	Netherlands	1.1
6	University of California, Davis	United States of America	1.1
7	King’s College London	United Kingdom	1.1
8	University of Toronto	Canada	1.1
9	Columbia University	United States of America	1.0
			10.7

**Table 2:** Journals from the research nucleus in the study of autism spectrum disorder.

Entry	Journal	Publisher	Impact Factor 2024	Q ^a^	Category ^b^	Per ^c^	P.T. ^d^
1	Journal of Child and Adolescent Psychopharmacology	Mary Ann Liebert, Inc.	2.2	Q2	Pediatrics	9.53	Hy
2	Progress in Neuro-Psychopharmacology & Biological Psychiatry	Pergamon-ElsevierScience Ltd.	3.9	Q1	Clinical Neurology/Pharmacology and Pharmacy/Psychiatry	8.15	Hy
3	Neuropsychopharmacology	Springer-Nature	7.1	Q1	Neurosciences/Pharmacology and Pharmacy/Psychiatry	6.27	OA
4	Neuropharmacology	Pergamon-ElsevierScience Ltd.	4.6	Q1	Neurosciences/Pharmacology and Pharmacy	4.89	Hy
5	Annales Medico-Psychologiques	Masson Editeur	0.5	Q4	Psychiatry/Psychology	4.81	Hy

^a^ Q = Best quartile reported by Web of Science according to 2024 statistics. ^b^ Category = Category corresponds to the best quartile reported by Web of Science according to 2024 statistics. ^c^ Per = Percentage according to the total number of articles analyzed (1170 published articles). ^d^ P.T. = Publication type: Subscription (S), Hybrid (Hy), or Open Access (OA).

**Table 3:** Characterization of the document corpus to be analyzed.

Variable	Value (or Sample, n)	Unit	Subsampling Criterion
Documents	1170	Article	Hirsch’s index (h-index)
Time	2001–2025	Year	Period without blanks, Price’s Law ^(1)^
Place (Affiliation)	68	Country/Territory	Census
Authors	6492	Person	Lotka’s Law
Keywords and Keywords Plus	2838 and 3244	Words	Zipf’s Law
Journals	187	Journal	Bradford’s Law

^(1)^ Price’s laws allow us to examine the exponential growth of science, measured.

## Data Availability

The original contributions presented in this study are included in the article/Supplementary Material. Further inquiries can be directed to the corresponding authors.

## Supplementary Materials

The following supporting information can be downloaded at: https://www.mdpi.com/article/10.3390/ph19010102/s1, Table S1: Institutions that contribute most to research related to the Autism Spectrum Disorder focused on the pharmacology & pharmacy field. ZIP compressed file containing the complete database exported from Web of Science in txt format for reading by VOSviewer.
