# Supplementary material for: Mapping Global Research Trends on Autism Spectrum Disorder: A Bibliometric Analysis of Pharmacology and Pharmacy Studies
Source: Pharmaceuticals (Basel). 2026 Jan 7;19(1):102. doi: 10.3390/ph19010102 (PMC12844977; doi:10.3390/ph19010102)
Supplement: Supplementary file 1 [file pharmaceuticals-19-00102-s001.zip › Pharm_Supplementary_Materials.pdf]

SUPPLEMENTARY MATERIALS

Autism Spectrum Disorders: Insights from Pharmacology and Pharmacy

Gianfranco Sabadini[1], Angelina Palacios-Muñoz[2,3], Isaac E. García[2,3], Javier Romero-Parra[4], Daniel Moraga[5], Mauricio Soto[6], Alejandro Vega-Muñoz[7, 8], Nicolás Contreras-Barraza[9], Guido Salazar-Sepúlveda[10, 11], Jaime Mella[1,12*], Marco Mellado[13*]

[1] Instituto de Química, Facultad de Ciencias, Universidad de Valparaíso, Valparaíso 2360102, Chile; gianfranco.sabadini@postgrado.uv.cl (G.S.), jaime.mella@uv.cl (J.M.).
[2] Centro de investigación en Ciencias Odontológicas y Médicas, Facultad de Odontología, Universidad de Valparaíso, Valparaíso 2360004, Chile; angelina.palacios@uv.cl (A.P.-M.), isaac.garcia@uv.cl (I.E.G.)
[3] Instituto Milenio Centro Interdisciplinario de Investigación en Neurociencia de Valparaíso, Universidad de Valparaíso, Valparaíso 2381850, Chile.
[4] Organic Chemistry and Physical Chemistry Department, Faculty of Chemical and Pharmaceutical Sciences, Universidad de Chile, Olivos 1007, Santiago 7820436, Chile; javier.romero@ciq.uchile.cl
[5] Laboratorio de Fisiología, Departamento de Ciencias Biomédicas, Facultad de Medicina, Universidad de Tarapacá, Arica, 1000000, Chile; dmoraga@academicos.uta.cl
[6] Departamento de Química, Universidad Técnica Federico Santa María, Av. España 1680, Valparaíso 234000, Chile; mauricio.soto@usm.cl
[7] Laboratorio de Bienestar y Comportamiento Organizacional, Universidad Central de Chile, Santiago 8330507, Chile; alejandro.vega@ucentral.cl
[8] Facultad de Ciencias Empresariales, Universidad Arturo Prat, Santiago 8340232, Chile.
[9] Facultad de Ciencias Económicas y Administrativas, Pontificia Universidad Católica de Valparaíso, Valparaíso 2340025, Chile; nicolas.contreras@pucv.cl
[10] Facultad de Ingeniería, Universidad Católica de la Santísima Concepción, Concepción 4090541, Chile; gsalazar@ucsc.cl
[11] Facultad de Ingeniería y Negocios, Universidad de Las Américas, Concepción 4090940, Chile.
[12] Centro de Investigación, Desarrollo e Innovación de Productos Bioactivos (CInBIO), Universidad de Valparaíso, Valparaíso 2360102, Chile
[13] Centro de Investigación en Ingeniería de Materiales, Universidad Central de Chile, Santiago 8330507, Chile; marco.mellado@ucentral.cl
* Correspondence: jaime.mella@uv.cl (J.M.); marco.mellado@ucentral.cl (M.M.).

Table S1: Institutions that contribute most to research related to the Autism Spectrum Disorder focused on the Pharmacology & Pharmacy field.

Entry	Institution	Country	Percentage of Contribution
**1**	King Saud University	Saudi Arabia	1.6
**2**	Harvard Medical School	United State of America	1.4
**3**	The Ohio State University	United State of America	1.2
**4**	Massachusetts General Hospital	United State of America	1.1
**5**	Radboud University Nijmegen	Netherlands	1.1
**6**	University of California, Davis	United State of America	1.1
**7**	King's College London	United Kingdom	1.1
**8**	University of Toronto	Canada	1.1
**9**	Columbia University	United State of America	1.0
**10**	Emory University	United State of America	0.9
**11**	Vanderbilt University	United State of America	0.9
**12**	National Institute of Mental Health	United State of America	0.9
**13**	University of Groningen	Netherlands	0.9
**14**	Osaka University	Japan	0.8
**15**	Stanford University	United State of America	0.7
**16**	University of Illinois	United State of America	0.7
**17**	Al-Azhar University	Egypt	0.7
**18**	Cincinnati Children's Hospital Medical Center	United State of America	0.7
**19**	Harvard University	United State of America	0.7
**20**	Konkuk University	South Korea	0.7
**21**	University of California, Los Angeles	United State of America	0.6
**22**	Johns Hopkins University	United State of America	0.6
**23**	University of Cincinnati	United State of America	0.6
**24**	University of Rochester	United State of America	0.6
**25**	Aarhus University	Denmark	0.6
**26**	University of Pittsburgh	United State of America	0.6
**27**	Tehran University of Medical Sciences	Iran	0.6
**28**	University of California, San Francisco	United State of America	0.4
**29**	Icahn School of Medicine at Mount Sinai	United State of America	0.4
**30**	Seoul National University	South Korea	0.4
**31**	Mahidol University	Thailand	0.4
**32**	Utrecht University	Netherlands	0.4
**33**	Harbin Medical University	China	0.4
**34**	University of Fukui	Japan	0.4
**35**	Yale University	United State of America	0.4
**36**	Peking University	China	0.4
**37**	Centre for Addiction and Mental Health	Canada	0.4
**38**	Ramathibodi Hospital, Mahidol University	Thailand	0.4
**39**	University of Missouri	United State of America	0.4

**40**	Nanjing Medical University	China	0.4
**41**	Boston Children's Hospital	United State of America	0.4
**42**	Sapienza University of Rome	Italy	0.4
**43**	University Medical Center Utrecht	Netherlands	0.4
**44**	University of North Carolina at Chapel Hill	United State of America	0.4
**45**	University of Electronic Science and Technology of China	China	0.4
**46**	United Arab Emirates University	United Arab Emirates	0.4
**47**	Ain Shams University	Egypt	0.4
**48**	Université Paris Cité (ex Université Paris Descartes, Paris V)	France	0.4
**49**	Istanbul University	Turkey	0.4
**50**	Tohoku University	Japan	0.4
		**Total**	**32.7**
